# A content analysis of internet information sources on medical cannabis

**DOI:** 10.1186/s42238-020-00041-1

**Published:** 2020-09-18

**Authors:** Daniel J. Kruger, Ilana M. Moffet, Liliah C. Seluk, Lara A. Zammit

**Affiliations:** 1 Population Studies Center, Institute for Social Research, University of Michigan, 426 Thompson St, Ann Arbor, MI 48109-1248 USA; 2 Literature, Science, and the Arts, University of Michigan, Ann Arbor, MI USA; 3 Undergraduate Research Opportunities Program, University of Michigan, Ann Arbor, MI USA

**Keywords:** Medical cannabis, Marijuana, Cannabinoid, Knowledge, Internet, Information

## Abstract

**Background:**

Medical cannabis users report that their knowledge regarding cannabis is predominantly from their own personal experiences and the Internet.

**Objective:**

We summarize and describe information found through Internet searches on medical cannabis in English language websites.

**Methods:**

We used terms related to medical cannabis in the Google search engine between November and December 2019. Resulting websites were catalogued and coded for content, including mentions of health and medical conditions, pharmacology, dosage, harmful or adverse effects, harm reduction techniques, cautions or warnings, products for sale, and credentials.

**Results:**

We coded 344 web pages on 179 unique websites. Cannabis was mentioned for the treatment of 151 different medical and health conditions, only four of the twenty most frequently mentioned conditions have received substantial empirical support for cannabis or cannabinoid treatment. Information content varied widely across sites, only a small proportion of sites included information on pharmacology, dosage, risks, and other aspects that are requirements for pharmaceutical drugs. Information provided was only moderately related to conclusions in the emerging scientific literature.

**Conclusions:**

Given the rise in cannabis use within the U.S. and the reliance on the Internet as a source of information, considerable efforts are needed to provide accurate on-line cannabis education to minimize harms and maximize benefits for individuals and society.

## Background

Cannabis is rapidly transitioning from an illicit substance to one legally available and widely used for both recreational and medicinal purposes. Changes in cannabis’ legal status have outpaced scientific and educational efforts, and regulatory requirements lag behind those for other psychoactive substances. In comparison, the Tobacco Control Act (Public Law 111–31 [H.R. 1256]) gives the U.S. Food and Drug Administration broad authority to regulate tobacco products, including requiring manufacturers to publish an understandable list of harmful and potentially harmful constituents and conducting consumer research to ensure that this list is not misleading. This Act also provides detailed requirements for the content and format of warning labels, with specific content required for different kinds of tobacco products. The Alcoholic Beverage Labeling Act of the Anti-Drug Abuse Act of 1988 (Public Law 100–690, 102 Stat. 4181 [H.R. 5210]) requires the labels of alcoholic beverages to carry specific government warnings against the use of alcohol during pregnancy because of the risk of birth defects, impairments when driving a car and operating machinery, and causing health problems. U.S. Food and Drug Administration’s Code of Federal Regulations Title 21 provides extensive requirements for the labeling of prescription and over-the-counter pharmaceutical drugs, including statements on identity, net quantity of contents, dosage, warnings regarding use during pregnancy and breastfeeding, etc. There are no such universal requirements for cannabis labeling or advertising, regulations vary at the state level. For U.S. states that have legalized medical cannabis, implementing comprehensive educational programs for patients, health professionals, and the general public has been a prominent challenge (Lamonica et al. 2016). Regulations about the content of educational materials are often vague and general, as are the educational materials in medical cannabis dispensaries (Lamonica et al. 2016).

Although 33 U.S. states currently permit the medical use of cannabis, only three currently require certification for physicians to recommend medical cannabis, which may entail only a couple of hours of training (Lombardi et al. 2020). Many physicians in a state with a medical cannabis program reported little knowledge of critical issues, such as the medical cannabis formulations available (Sideris et al. 2018). Canadian nurse practitioners also reported considerable gaps in cannabis knowledge and considered this lack of necessary information to be a barrier to authorizing medical cannabis (Balneaves et al. 2018). Many physicians have negative attitudes towards cannabis and are not likely to recommend it to their patients, even when in a state with legal medical access (Lombardi et al. 2020). Public health education efforts regarding cannabis have been limited in scope, with a typical focus on adverse effects and abstinence promotion, a legacy from the era of cannabis prohibition. More recently, efforts such as Colorado’s Good to Know and Responsibility Grows Here campaigns (Brooks-Russell et al. 2017) and the Drug Policy Alliance’s Safety First: Real Drug Education for Teens (http://www.drugpolicy.org/) have focused on harm reduction with an emphasis on providing accurate information.

Understanding of cannabis is generally modest among the general public (e.g., Zeiger et al. 2020). Medical cannabis users have remarkably low to moderate knowledge of important aspects of cannabis such as medical effectiveness (Kruger et al. 2020a), cannabinoid content and effective dosages (Kruger et al. 2020b), and effective harm reduction techniques (Kruger et al. 2020c). Those who use cannabis medicinally report both trusting and using medical cannabis considerably more so than mainstream healthcare (Kruger and Kruger 2019), and thus may not seek cannabis-related information from mainstream healthcare providers. Medical cannabis users report that their knowledge regarding cannabis is primarily obtained from their own personal experiences, and websites on the Internet are the most prevalent external sources of information (Kruger et al. 2020b). Internet-based social media are often used as a mechanism to share information about cannabis, including links to informational websites and media reports (Dakkak et al. 2018).

The lack of integration between mainstream health care and the medical use of cannabis can result in problematic issues, especially given the low level of cannabis knowledge among both health care professionals and those who use cannabis medicinally. For example, many of those who use cannabis to treat health or medical conditions will reduce or replace their use of prescription drugs (Reiman 2009; Reinarman et al. 2011). In many cases, their primary care provider is unaware of this substitution (Kruger and Kruger 2019). Similarly, cannabis is known to have adverse interactions with some pharmaceutical drugs (Cox et al. 2019), presenting dangers for lack of integrated care.

Given the importance of issues related to the medical use of cannabis and the reliance on the Internet as an information source by medicinal users, it would be valuable to characterize the information encountered in a naturalistic Internet search, and also to compare this information with current conclusions based on empirical evidence. The project described here addresses these needs.

## Methods

We used the Google search engine with the search terms “medical cannabis,” “types of medical marijuana,” “medical marijuana,” “cannabis for medicine,” “medical cannabis for,” “cannabis and health,” and “marijuana and health” between November and December 2019. Resulting websites were catalogued and we continued searching until no new websites were found. Pages with abstracts for non-open access journal articles were excluded. Different pages found on the same website were coded separately. We coded the sites for content, recording: The date the website was accessed; any publication date given; whether the site included age verification; and health or medical conditions that cannabis was recommended for treating. We coded whether or not the website mentioned specific cannabis strains or products, THC levels, CBD levels, other cannabinoids or molecules such as terpenes, harmful or adverse effects, harm reduction techniques, cautions or warnings, products for sale, credentials of content author, and citations of external sources.

## Results

We coded 344 pages on 179 unique websites. Publication dates were provided on 57% of websites, ranging from 10/14/2010 to 11/13/2019. Only a few (3%) sites had age verification click boxes, none had any stronger form of age verification (e.g., credit card number entry). Most (92%) sites mentioned a specific health or medical condition that could be treated with cannabis. Cannabis was mentioned for the treatment of 151 different medical and health conditions (See Table 1). These included conditions for which the National Academies of Sciences, Engineering, and Medicine (NASEM 2017) determined that there is conclusive or substantial evidence that cannabis or cannabinoids are effective in treatment (4% of conditions mentioned), conditions with moderate evidence for treatment effectiveness (1%), conditions for which evidence for effectiveness was limited (6%), conditions with no or insufficient evidence for treatment effectiveness (9%), conditions with evidence for the ineffectiveness of treatment (3%), and conditions with evidence for increased risk due to the use of cannabis (3%). Most (74%) conditions mentioned were not addressed by the NASEM report, especially those mentioned by only one Internet source (94% of conditions on 78 web pages).

**Table 1:** Health and medical conditions recommended for cannabis treatment on 179 identified websites and corresponding conclusions from the National Academies 2017 review

Condition	Percent	NASEM conclusion
Nausea (including from chemotherapy)	26	Conclusive or substantial
Pain	24	(no conclusion)
Epilepsy	23	None or insufficient
Multiple sclerosis	21	Conclusive or substantial
Chronic pain	20	Conclusive or substantial
Anxiety	20	Limited
Appetite loss	19	Limited
Cancer	19	None or insufficient
Glaucoma/Intraocular pressure	15	Ineffective (limited)
Depression	14	Ineffective (limited)
Vomiting (including from chemotherapy)	14	Conclusive or substantial
Chronic Fatigue Syndrome	13	(no conclusion)
Insomnia	13	(no conclusion)
PTSD	13	Limited
Neuropathic pain	11	(no conclusion)
Arthritis (including various forms)	10	(no conclusion)
Inflammation	10	Limited
HIV/AIDS	10	None or insufficient
Muscle spasms	10	(no conclusion)
Drug and alcohol addiction	9	Increased risk (moderate)

Note: NASEM Conclusion indicates determinations by the National Academies of Sciences, Engineering, and Medicine (2017) that there is conclusive or substantial evidence that cannabis or cannabinoids are effective in treatment, conditions for which evidence for effectiveness was limited, conditions with no or insufficient evidence for treatment effectiveness (9%), conditions with evidence for the ineffectiveness of treatment, and conditions with evidence for increased risk due to the use of cannabis

Most (66%) sites mentioned specific cannabis strains or products, 13% of sites mentioned THC levels, 7% of sites mentioned CBD levels, and 4% of sites mentioned levels of other cannabinoids or terpenes. A small percentage of sites (6%) listed products for sale. Prescription cannabinoid products mentioned included Marinol/dronabinol (*n* = 39, 11% of sites), Cesamet/nabilone (*n* = 32, 9%), Sativex/nabiximol (*n* = 25, 7%), and Epidiolex (*n* = 25, 7%). Some sites mentioned indica, sativa, or hybrid strains in general, others mentioned specific cultivars such as Maui wowie, pineapple express, and Jack Herer. Charlotte’s Web was mentioned by several sites as an example of a strain with low THC. Sites mentioned THC and CBD in several contexts. Some provided concentrations for particular strains, several mentioned 0.3% as the definition for low-THC strains (classified as hemp), several gave recommendations for specific dosages for specific conditions, several mentioned the importance of considering ratios of THC to CBD, and one mentioned that 10 mg of THC was considered one serving. Some sites mentioned information such as the historical rise in THC level or that high THC strains can increase anxiety.

About one-third (30%) of sites mentioned harmful or adverse effects, including conditions identified by the NASEM (2017) as having substantial evidence of association with cannabis use (heightened risk for the development of schizophrenia or other psychoses, increased risk of motor vehicle accidents), many experiences commonly associated with cannabis use (altered sensations and perceptions, dry mouth, anxiety, hunger, paranoia, impairments in cognition and short-term memory), and other adverse effects which have not yet been substantiated (harm to immune system, increased risk of bleeding, harm to blood vessels, heart attacks).

Fourteen sites (4%) mentioned harm reduction techniques, including some of those identified by Fischer et al. (2017) as effective (do not smoke/use a vaporizer, do not operate machinery or drive a vehicle after consuming, avoid use while pregnant) and others which are common and useful advice across many types of drugs (avoid using cannabis with alcohol and other drugs, know what you are using and how it can affect you, consider the potential drug interactions when taking other medications). One-fifth of (20%) sites mentioned cautions or warnings, with a wide range of content. These included legal status and regulations, the historical increase in cannabinoid concentrations, the lack of regulatory oversight of CBD oils, adverse side effects, potential risks (including dependency, illnesses, injury, and death), lack of knowledge regarding cannabis, and advice to consult with a physician before making changes in the use of medications.

One-tenth (10%) of sites listed credentials for the article’s author. Authors’ credentials included “MD” (*n* = 19, 6%), “PhD” (*n* = 4, 1%), “PharmD” (*n* = 3, 1%), other academic or professional degrees (*n* = 3, 1%; MA, MPH, PsyD), and “RN” (*n* = 1). Two sites listed ambiguous credentials, “Dr.” and “Marijuana Doctors.” Some (8%) pages indicated that they were “medically reviewed,” often providing the name of the reviewer and their credentials (e.g., MD, PhD, MA, or PharmD). A few (6%) sites included citations or indications of the source of the information provided. Some (*n* = 13, 4%) of these citations were of peer reviewed journal articles, others referenced government agencies or entities (U.S. National Institutes of Health, U.S. Food and Drug Administration, U.S. Drug Enforcement Administration, U.S. National Institute on Drug Abuse, U.S. National Library of Medicine, U.S. National Academies of Sciences), a few referenced other websites (Drugs.com, Wikipedia.org).

## Discussion

The accessibility of cannabis is rapidly increasing in the U.S. and many other countries. This increases the importance of promoting the public’s understanding of cannabis related issues. The potential for misuse is higher when there is a lack of accurate information, especially for those who are self-medicating. The Internet is a frequent source for information, including for those seeking information on the medical use of cannabis (Kruger et al. 2020b). This may be in part a legacy of the historical criminalization of cannabis, which prevented integration with the mainstream health care system and resulted in the proliferation of anti-cannabis use themed health promotion advertisements, which now may be seen as factually inaccurate. On-line information is extensive, yet there is little regulation of content. Websites are rarely peer-reviewed or otherwise vetted for accuracy.

The current study found that information related to medical cannabis on the Internet is abundant, generally positive, but lacking in the depth and detail that would ideally inform medical treatment. Over 150 different health and medical conditions were suggested for treatment by cannabis, yet only 4% of the conditions mentioned have substantial empirical support (NASEM 2017). Advocacy based on non-empirical information could promote inappropriate use of cannabis to treat medical conditions and may undermine the use of non-cannabis treatment(s) that are more effective.

Websites were three times as likely to mention cannabis for the treatment of a health or medical condition as they were to mention harmful or adverse effects. Cannabis may pose dangers to pregnant and breastfeeding women (Thompson et al. 2019). For example, cannabis use during pregnancy triples the likelihood of having a low birth weight baby, even after adjusting for factors such as socioeconomic status, medical history, and other substance use such as tobacco smoking (Campbell et al. 2018). The increasing levels of THC content in cannabis also raises concerns for cannabis use while breastfeeding (Seabrook et al. 2017). A minority mentioned cautions or warnings, and even fewer mentioned harm reduction techniques. Harm reduction strategies with empirical support include vaping rather than smoking, using cannabis strains with high cannabidiol (CBD) to tetrahydrocannabinol (THC) ratios, and avoiding driving within 6 hours of using cannabis, mixing cannabis with tobacco, or using when pregnant (Fischer et al. 2017).

Previous research indicates that substantial proportions of cannabis users report acute adverse reactions, which may be more frequent among less experienced users who have limited knowledge (LaFrance et al. 2020). Few websites mentioned even the most commonly known cannabinoids, THC and CBD, and only a handful of these sites recommended specific dosages for particular conditions. Although there are no universally accepted guidelines for cannabinoid dosages, existing guidelines recommend between 2 mg and 10 mg of THC (Freeman and Lorenzetti 2020; Sideris et al. 2018; State of California Senate 2017) and titrations of CBD in 2 mg increments (Canadian Pharmacists’ Association 2019). Most sites did not provide details on the information sources or the credentials of those authoring the articles.

Given the current levels of knowledge regarding medical cannabis and the state of information readily available through the Internet, it would be very useful to provide a holistic and integrated source of empirically supported information accessible through the Internet and understandable to the general public. The U.S. Food and Drug Administration provides a comprehensive resource on-line for regulatory requirements of pharmaceuticals, identifying what information needs to be provided, though not providing the information specific to each pharmaceutical. The National Academies of Sciences, Engineering, and Medicine (NASEM) 2017 report, *The health effects of cannabis and cannabinoids,* may be the most comprehensive report to date and is available on-line, along with a summary of the conclusions. Given the rapid legalization of medicinal cannabis (currently at the state level), the industrialization of the cannabis industry, the large and growing population of medicinal cannabis users, and the prevalence of the Internet as an information source, it would be very valuable for the National Academies to provide updates of findings on a frequent basis through the Internet. Providing a trusted source of vetted information may be most effective at maximizing benefits and minimizing risks among medical cannabis users. Given the current scientific understanding of cannabis, it would be valuable to honestly and accurately highlight what is currently uncertain or unknown, describing issues which should be clarified with future research, such as condition-specific cannabinoid dosages.

Other strategies may complement the establishment of a centralized information source. Open-access peer-reviewed journals are available to the public. It would be helpful to convey the implications of research findings, highlighted in ways understandable to a wide audience. Even non-open access journals could include open-access highlights to disseminate research findings. Research is often covered by media and other secondary sources and research on medical cannabis may generate particular interest. Researchers could develop media releases or other public statements that describe their studies and findings in intuitive ways so that implications are not truncated or distorted when described by others.

Of course, considerable research efforts are needed to provide a full understanding of the medicinal use of cannabis. Systematic research is needed on treatment effectiveness for various health conditions; the effective dosage levels for the numerous cannabinoids by health condition; optimal administration forms and schedules; health and other risks; effectiveness of harm reduction techniques; and the effectiveness of health education techniques custom-tailored for cannabis users. In the Federal Controlled Substances Act (Pub. L. 91–513, title II, § 202, Oct. 27, 1970), the U.S. government classified cannabis as a Schedule I drug, grouped with substances determined to have no accepted medical use and a high potential for abuse. No prescriptions may be written for Schedule I substances and legal production is restricted. These conditions interfere with research needs, for example the double-blind randomized placebo-controlled studies which are the gold standard for pharmaceutical drug trials. Schedule I status may also be a barrier to the research funding which is critical to a comprehensive research program which would inform educational efforts.

### Limitations

The research team attempted an exhaustive search, however information on the Internet is subject to change and may differ by search methodology. Academic journals were generally excluded, as the project goal was to document the information available to the general public, rather than those who have university library access. English words were used as search terms, non-English language websites were not coded. We did not systematically record whether or not websites recommended using cannabis as a substitute or replacement for pharmaceutical drugs, however coders reported not noticing such recommendations. It is possible that websites would not make such recommendations due to liability concerns.

## Conclusions

Reliance on the Internet for information on medical cannabis is an indication of the current lack of integration with mainstream healthcare. The information obtained through a typical Internet search bears little resemblance to findings in the current scientific literature, let alone a comprehensive guide to best practices informed by empirical evidence. Given the rise in cannabis use within the U.S. and the reliance on the Internet as a source of information, considerable efforts are needed to provide comprehensive on-line cannabis education to minimize harms and maximize benefits for individuals and society. Educational efforts need to be informed by a comprehensive and well-funded research program addressing areas where conclusions are currently unavailable.

## Data Availability

The dataset used and analyzed for the current study are available from the corresponding author on reasonable request.

## Abbreviations

THC: Tetrahydrocannabinol
CBD: Cannabidiol
NASEM: National Academies of Sciences, Engineering, and Medicine
U.S.: United States
PTSD: Post-traumatic stress disorder
MD: Doctor of Medicine
PhD: Doctor of Philosophy
PharmD: Doctor of Pharmacy
MA: Master of Arts
MPH: Master of Public Health
PsyD: Doctor of Psychology
RN: Registered Nurse
